# Disparities in direct acting antivirals uptake in HIV‐hepatitis C co‐infected populations in Canada

**DOI:** 10.1002/jia2.25013

**Published:** 2017-11-08

**Authors:** Sahar Saeed, Erin C Strumpf, Erica EM Moodie, Jim Young, Roy Nitulescu, Joseph Cox, Alexander Wong, Sharon Walmsely, Curtis Cooper, Marie‐Lousie Vachon, Valerie Martel‐Laferriere, Mark Hull, Brian Conway, Marina B Klein, the Canadian Co‐Infection Cohort Study

**Affiliations:** ^1^ Department of Epidemiology, Biostatistics, and Occupational Health McGill University Montreal QC Canada; ^2^ Division of Infectious Diseases/Chronic Viral Illness Service Department of Medicine Glen site McGill University Health Centre Montreal QC Canada; ^3^ Department of Economics McGill University Montreal QC Canada; ^4^ Regina Qu'Appelle Health Region Regina SK Canada; ^5^ University Health Network Toronto ON Canada; ^6^ CIHR Canadian HIV Trials Network Vancouver BC Canada; ^7^ Department of Medicine University of Ottawa Ottawa ON Canada; ^8^ Centre Hospitalier de l'Université Laval Quebec QC Canada; ^9^ Centre de Recherche du Centre hospitalier de l'Université de Montréal Montreal QC Canada; ^10^ Centre for Excellence in HIV/AIDS St. Paul's Hospital Vancouver BC Canada; ^11^ Vancouver Infectious Diseases Centre Vancouver BC Canada

**Keywords:** HIV‐hepatitis C co‐infection, Direct acting antivirals (DAAs), People who inject drug (PWID), Disparities, Indigenous peoples, Women, Men who have sex with men (MSM)

## Abstract

**Background:**

Direct acting antivirals (DAAs) have revolutionized hepatitis C (HCV) treatment with >90% cure rates even in real‐world studies, giving hope that HCV can be eliminated. However, for DAAs to have a population‐level impact on the burden of HCV disease, treatment uptake needs to be expanded. We investigated temporal trends in HCV treatment uptake and evaluated factors associated with second‐generation DAA initiation and efficacy among key HIV‐HCV co‐infected populations in Canada.

**Methods:**

The Canadian HIV‐HCV Co‐Infection Cohort Study prospectively follows 1699 participants from 18 centres. Among HCV RNA+ participants, we determined the incidence of HCV treatment initiation per year overall and by key populations between 2007 and 2015. Key populations were based on World Health Organization (WHO) guidelines including: people who actively inject drugs (PWID) (reporting injection drug use, last 6 months); Indigenous people; women and men who have sex with men (MSM). Multivariate Cox models were used to estimate adjusted hazard ratios (aHR) and 2‐year probability of initiating second‐generation DAAs for each of the key populations.

**Results:**

Overall, HCV treatment initiation rates increased from 8 (95% CI, 6–11) /100 person‐years in 2013 to 28 (95% CI, 23–33) /100 person‐years in 2015. Among 911 HCV RNA + participants, there were 202 second‐generation DAA initiations (93% with interferon‐free regimens). After adjustment (aHR, 95% CI), active PWID (0.60, 0.38–0.94 compared to people not injecting drugs) and more generally, people with lower income (<$18 000 CAD/year) (0.50, 0.35, 0.71) were less likely to initiate treatment. Conversely, MSM were more likely to initiate 1.95 (1.33, 2.86) compared to heterosexual men. In our cohort, the population profile with the lowest 2‐year probability of initiating DAAs was Indigenous, women who inject drugs (5%, 95% CI 3–8%). Not having any of these risk factors resulted in a 35% (95% CI 32–38%) probability of initiating DAA treatment. Sustained virologic response (SVR) rates were >82% in all key populations.

**Conclusion:**

While treatment uptake has increased with the availability of second‐generation DAAs, marginalized populations, already engaged in care, are still failing to access treatment. Targeted strategies to address barriers are needed to avoid further health inequities and to maximize the public health impact of DAAs.

## 1 Introduction

Broad access to combination antiviral therapy (cART) led to tremendous improvements to the lives of people living with human immunodeficiency virus (HIV), including dramatic reductions of acquired immune deficiency syndrome (AIDS) related morbidity and mortality 1, 2. However, despite controlled HIV viraemia and immune restoration, liver disease has now emerged as a leading cause of death among HIV‐positive individuals largely due to Hepatitis C virus (HCV) coinfection 3. HIV‐HCV coinfection affects approximately 2.3 million people worldwide and represents a particular challenge in Eastern Europe and Russia, in Indigenous communities in Canada and in rural North America where injection drug use drives the emerging epidemic 4, 5, 6.

The development of oral direct acting antivirals (DAAs) revolutionized HCV treatment with over 90% cure rates even in real‐world settings, giving hope that HCV can be eliminated 7, 8, 9, 10, 11, 12, 13. However, for DAAs to have a population‐level impact on the burden of HCV disease, treatment uptake needs to be expanded 14, 15. Historically, HCV treatment uptake in North America and Europe among HIV‐HCV coinfected individuals was as low as 1% 16, 17, 18, 19, 20, 21. This is particularly true among people who inject drugs (PWID), a key population to target if the goal is to reduce incident HCV infections 14, 22.

Barriers to accessing HCV treatment emerge at each step of the HCV care continuum 23. A combination of patient‐, provider‐ and system‐level barriers have previously been identified as reasons why patients fail to access treatment 20. Patient‐level barriers include competing priorities, lack of awareness and co‐morbidities 21. Preconceived fears of poor adherence and risk of reinfection have been reported as reason for provider‐level barriers 21. Although improved efficacy and tolerability of DAAs have addressed many clinical barriers to treatment initiation, these have largely been replaced by financial ones 24. Indeed in many countries, financial barriers are the principle reasons for reduced access to HCV therapy. Since Canada's healthcare system is publicly funded, it should be less driven by an individual's ability to pay, however, other factors such as lower socio‐economic status (SES) and Indigenous status, have been associated with health disparities 25, 26. The extraordinary cost of DAAs has led to policies restricting access to HCV treatments worldwide, resulting in system‐level barriers 27, 28, 29. Despite international guidelines to treat “all” populations infected with HCV 30, considerable variability in DAA reimbursement exists 13. Reimbursement of DAAs in Canada, varies across provinces by liver disease stage, HCV genotype and prescriber type 27. While in other jurisdictions such as in the United States, patient characteristics such as illicit drug and alcohol use are used as restrictions, due to concerns about potential for non‐adherence and reinfection 29.

Currently limited data exist on treatment initiation rates among key HIV‐HCV coinfected populations. The World Health Organization (WHO) defines key populations as those “most‐at‐risk of HIV and viral hepatitis transmission” which include PWID and men who have sex with men (MSM) in addition to country specific populations considered to be vulnerable 31. In Canada, Indigenous peoples are almost three times more likely to acquire HIV compared with other Canadians and can therefore be considered a key population 32. Women may also face unique barriers to treatment and are often not enrolled in clinical trials. Developing strategies to treat all co‐infected populations is essential to both manage incident cases of HCV 14, 22, 33 and reduce morbidity and mortality in those at greatest risk for liver disease progression 34, 35. The purpose of this study was to investigate if disparities in HCV treatment initiation rates exist among key HIV‐HCV co‐infected populations already engaged in care and to identify factors associated with failure to initiate second‐generation DAAs.

## 2 Methods

### 2.1 Study population

The Canadian Co‐infection Cohort Study (CCC) is a publicly funded prospective cohort of 1699 HIV‐HCV coinfected individuals from across Canada 36. Enrolment of HIV‐positive adults with evidence of HCV infection (antibody positive) began in 2002. In 2006, the cohort expanded nationally and continues to recruit actively from 18 centres. Participating centres comprise of urban tertiary care and community‐based hospitals, private clinics and street outreach programs in the attempt to capture a representative sample of patients in care 36. After obtaining informed consent, socio‐demographic, behavioural and clinical data are collected prospectively via self‐administered questionnaires and chart review every 6 months. Since 2012 the main focus of the CCC has been to study the “real‐world” impact of DAAs on health outcomes in HIV‐HCV coinfection. Details on HCV treatments and subsequent responses are extracted from participant's medical records using standardized case report forms. The CCC is approved by the community advisory committee of the Canadian Institutes of Health Research (CIHR) Canadian HIV Trials Network and by all institutional ethics boards of participating centres.

#### 2.1.1 Key populations (main exposures)

Key populations were identified *a priori* based on WHO guidelines 31. Definitions were extracted from self‐reported data collected by semi‐annual questionnaires. Key populations included active PWID as defined as injection drug use within the last 6 month); Indigenous people of Canada defined as either people of First Nations, Inuit or Metis origins; women based on biological sex and MSM.

#### 2.1.2 Outcomes

All outcomes (defined below) were examined overall and by key populations of interest.

### 2.2 Temporal trends in HCV treatment initiation rates

Participants were potentially eligible to initiate any HCV treatment if they were both actively participating in the CCC (alive, with a cohort visit within 1 year) and HCV RNA positive. HCV RNA was measured in local laboratories using either a qualitative assay (COBAS^®^ Ampliprep/TaqMan^®^ HCV Test, v2.0, Roche Molecular Systems (Pleasanton, CA, USA), or other local laboratory assays; lower limit of detection varied by assay and year) or quantitative assay (Abbot RealTime PCR; Abbott Molecular Inc (Abbott Park, IL, USA), or other local laboratory assays; lower limit of detection varied by assay and year). HCV treatment initiation rates were calculated from January 1st 2007 until December 31st 2015.

### 2.3 Uptake of second‐generation DAAs

Second‐generation DAAs were defined as Health Canada approved regimens containing simeprevir, sofosbuvir, ledipasvir, ombitasvir/paritaprevir/ritonavir or daclatasvir. Participants were potentially eligible to initiate second‐generation DAAs if they were both HCV RNA positive as of November 21, 2013 (date Health Canada approved simeprevir) and had not accessed second‐generation DAAs through a clinical trial. Participants were followed until DAA initiation or censored if lost to follow‐up (no study visit for at least 1.5 years), died, withdrew or at the end of the study period (December 31st 2015).

### 2.4 Efficacy of second‐generation DAAs

Sustained virologic response (SVR) was defined as documented negative HCV RNA result at least 12 weeks after completing HCV treatment. SVRs results were determined up until December 31, 2016.

### 2.5 Statistical analysis

Hepatitis C virus treatment incidence rates were reported as 100 person‐years, by calendar year. Demographics, SES, illicit drug and alcohol consumption, HIV and HCV related treatments and clinical factors were compared between people who initiated second‐generation DAAs to those who did not initiate treatment. SVR rates were compared between key populations using Fisher's exact test.

We estimated unadjusted and adjusted hazard ratios (aHR) and 95% confidence intervals (CI) for time to second‐generation DAA initiation using Cox proportional hazards models. The adjusted Cox model included the key exposures of interest (indicators for Indigenous status, sex, active injection drug use and MSM) along with other predictors of treatment initiation selected *a priori*. Predictors included: (1) socio‐demographic – age (centred at mean) and income (<$18 000 CAD)^37^; (2) behavioural – past (but not current) injection drug use and current alcohol use (within the last 6 months); (3) clinical – HCV genotype 2, 3 or 4 compared to genotype 1, advanced fibrosis (measured as an AST to Platelet Ratio Index (APRI) score ≥1.5) and undetectable HIV RNA (<50 copies/mL); and (4) healthcare systems – Canadian province of residence (Saskatchewan, Alberta/Ontario and Quebec compared to British Columbia; grouped to reflect DAA policy restrictions 27). Robust standard errors were used to adjust for possible clustering by centre. Multiple imputation by chained equations was used to impute missing data on HCV genotype (89/712 were missing) in multivariate models 38. The imputation model included all covariates in the multivariable model, an indicator for DAA initiation, and a measure of the cumulative baseline hazard using the Nelson‐Aalen estimator. Twenty imputed data sets were created and Rubin's rules were used to combine regression results 39. Using the adjusted Cox model, the baseline survival function at 2 years was estimated using the post‐estimation command (*predict*) to calculate probabilities of second‐generation DAA initiation and 95% CI 40. The 2‐year probability of second‐generation DAA initiation was summarized graphically for the key groups of interest who were less likely to initiate treatment (combination of being Indigenous, a women and an active injection drug use). As a sensitivity analysis, stratified Cox models were evaluated independently for each of the key populations. Graphical methods were used to check the proportional hazards assumption of Cox models. All statistical analyses were performed using STATA version 13.

## 3 Results

As of September 30, 2016, 1699 participants had enrolled into the CCC. The median age of cohort participants at baseline was 45 years old (IQR 39, 51) and 81% had a history of injection drug use (IDU). Twenty‐eight percent were women, 21% were Indigenous and 23% were MSM.

### 3.1 Trends in overall HCV treatment initiation rates

**Figure 1:**
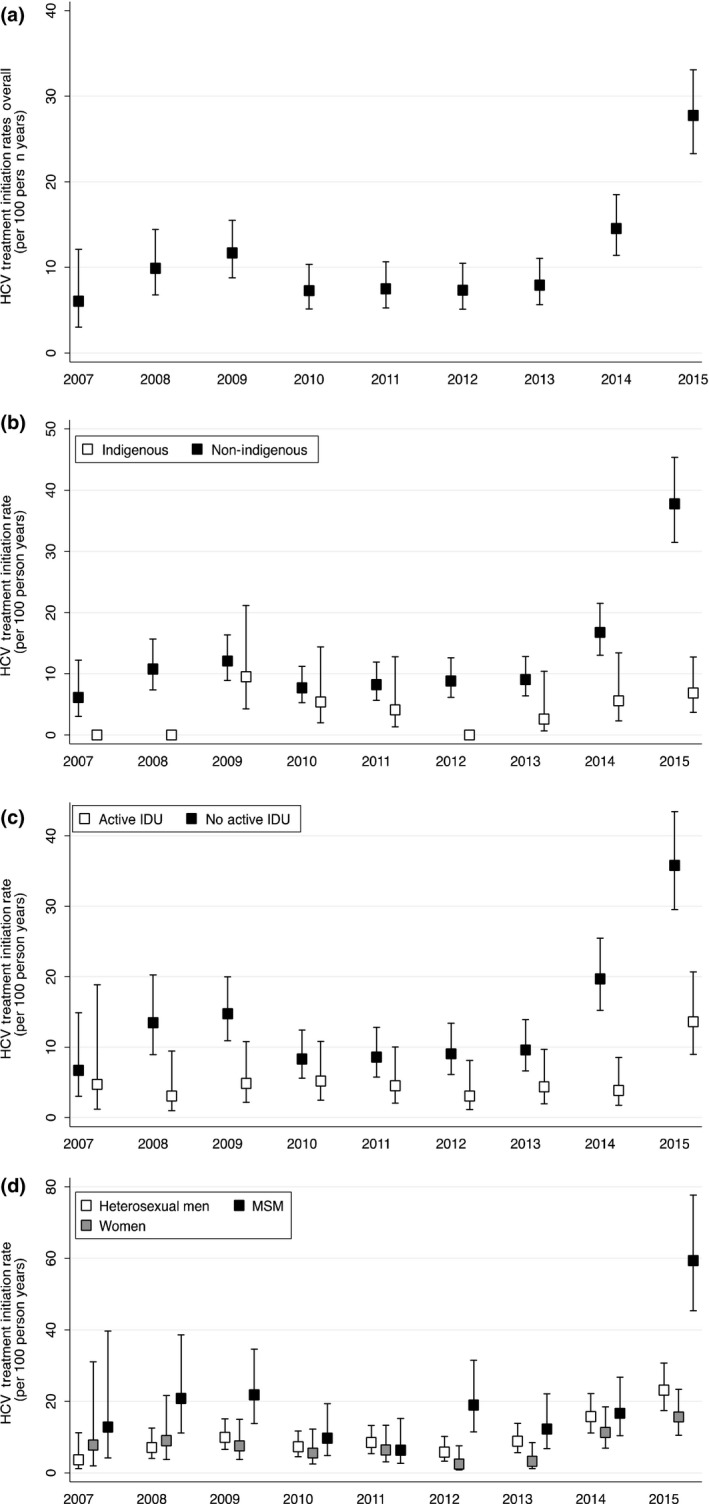
HCV Treatment Initiation Rates between 2007–2015. A: Overall among the Canadian Co‐Infection Cohort; B: Indigenous (white box) compared to Non‐Indigenous people (black box); C: Active PWID (white box) compared to non‐Active PWID (black box); D: Women (grey box) and MSM (black box) compared to heterosexual men (white box). Rates per 100 person years, whiskers represent 95% confidence intervals. PWID: people who inject drugs; MSM: men who have sex with men; IDU: injection drug use.

HCV treatment initiation rates remained relatively stable (5–11 initiations per 100 person‐years) between 2007 until 2013. With the introduction of second‐generation DAAs, initiation rates increased more than threefold between 2013 and 2015, from 8 (95% CI: 6, 11) to 28 (95% CI: 23, 33) per 100 person‐years (Figure 1, Panel A). After stratifying initiation rates by key populations, HCV treatment uptake was markedly lower among Indigenous peoples (Panel B), active PWID (Panel C) and women (Panel D) compared to non‐Indigenous peoples, non‐active PWID, heterosexual men respectively. Conversely, MSM (Panel D) initiated HCV treatment at a higher rate compared to heterosexual men.

### 3.2 Factors associated with second‐generation DAA initiation

The DAA treatment eligible cohort consisted of 911 participants (Figure S1). Characteristics of participants excluded from this analysis (those lost/withdrew before time zero and who accessed DAAs through a clinical trial) are summarized in Table S1. The median follow‐up time was 2.1 years (IQR 1.9–2.1). There were a total of 202 second‐generation DAAs initiations– three people initiated twice. Of the 712 participants who did not initiate DAAs, 120 participants were censored (83 (9%) were lost to follow‐up, 8 (<1%) withdrew and 29 (3%) died) and the remaining 592 participants were followed until the end of the study. Demographic, behavioural, HIV and HCV clinical characteristics of the 199 participants who initiated second‐generation DAAs were compared to the 712 who did not initiate (Table 1).

**Table 1:** Baseline characteristics of the Canadian Coinfection Cohort Participants who initiated second‐generation DAA treatments compared to those who did not

	Initiated DAA N = 199^a^^,^ b	Eligible for treatment but did not initiate DAAs N = 712^c^
Age, median (IQR), years	50 (47, 55)	47 (40, 53)
Women, n (%)	39 (19%)	228 (32%)
Indigenous people, n (%)	19 (9%)	228 (32%)
Men who have sex with men (MSM), n (%)	82 (41%)	121 (17%)
Single, n (%)	132 (65%)	490 (69%)
Education (>high school diploma), n (%)	79 (39%)	148 (21%)
Gross annual income^d^, <$18 000 CAN, n (%)	131 (65%)	569 (80%)
Canadian provinces 27, n (%)		
British Columbia	58 (29%)	198 (28%)
Saskatchewan	1 (<1%)	146 (21%)
Alberta	4 (2%)	17 (2%)
Ontario	50 (25%)	158 (22%)
Quebec	87 (43%)	192 (27%)
Nova Scotia	2 (1%)	1 (<1%)
Current psychiatric diagnosis, n (%)	39 (19%)	163 (23%)
Currently living in shelter or homeless, n (%)	14 (7%)	90 (13%)
Ever injection drug use (IDU), n (%)	144 (71%)	616 (87%)
Past PWID^e^, n (%)	105 (52%)	340 (48%)
Active PWID^f^, n (%)	39 (19%)	273 (38%)
Current alcohol use, n (%)	106 (53%)	387 (54%)
Current alcohol abuse^g^, n (%)	21 (10%)	150 (21%)
Current tobacco smokers, n (%)	161 (80%)	663 (93%)
Time since HIV diagnosis, median (IQR), (years)	17 (12, 23)	13 (7, 19)
Undetectable HIV RNA (<50 copies/ml), n (%)	174 (86%)	499 (70%)
CD4 T‐cell count, median (IQR), (cells/mm^3^)	440 (270, 630)	456 (269, 650)
On cART, n (%)	190 (94%)	604 (85%)
Duration HCV infection, median (IQR), years	22 (12, 31)	21 (12, 29)
HCV genotype, n (%)		
1	161 (80%)	467 (66%)
2	11 (5%)	28 (4%)
3	23 (11%)	119 (17%)
4	7 (4%)	9 (1%)
Missing	0	89 (13%)
Prior HCV treatment experience, n (%)	78 (39%)	85 (12%)
Missing	8 (4%)	
Current APRI >1.5, n (%)	71 (35%)	128 (18%)
History of ESLD diagnosis^h^, n (%)	78 (39%)	100 (14%)

Baseline/Current (refers to, time zero (November 2013)).

HCV: hepatitis C virus; IDU: injection drug use; PWID: person who injects drugs; cART: combined antiretroviral therapy; PI: protease inhibitors; HCV: hepatitis C virus; APRI: AST to platelet ratio index.

a Included the following regimens [133 initiations were with ledipasvir/sofosbuvir; 28 with sofosbuvir/ribavirin; 19 with sofosbuvir/ simeprevir +/− ribavirin; 13 with sofosbuvir/ribavirin/peg‐interferon; 4 with ombitasvir/paritaprevir/ ritonavir/ribavirin; 3 with sofosbuvir/daclatasvir and 2 simeprevir/ribavirin/peg‐interferon].

b 199 unique people initiated treatment, three people initiated twice (n = 202 initiations).

c Includes all active participants, with a positive HCV RNA result, who did not initiate DAAs (see Table S1 for details).

d Single person low income is considered annual income of <$18 421/yr CAN 37.

e Active PWID: Use of any injection drugs within 6 months of last cohort visit (self‐reported).

f Past PWID: Not actively injecting drugs (as defined above) however exposure to injection drugs while participating in the CCC study (self reported).

g Current Alcohol Abuse: Drinking more than 2 units of alcohol on a “typical day” within 6 months of last cohort visit (self reported).

h ESLD‐End Stage Liver Disease (clinical diagnosis of: ascites, bleeding oespohageal varices, portal hypertension, hepatocellular carcinoma, spontaneous bacterial peritonitis).

The vast majority of DAA regimens were interferon free (93%): 133 initiations were with ledipasvir/sofosbuvir; 28 with sofosbuvir/ribavirin; 19 with sofosbuvir/simeprevir +/− ribavirin; 13 with sofosbuvir/ribavirin/pegylated‐interferon; 4 with ombitasvir/paritaprevir/ritonavir/ribavirin; 3 with sofosbuvir/daclatasvir and 2 simeprevir/ribavirin/pegylated‐interferon. Those who initiated HCV treatment were less likely to be Indigenous, women and active PWID (Table 1). Participants who initiated HCV treatment were more likely to be MSM, have a gross annual income above the low‐income threshold 37, undetectable HIV viral load, more advanced liver disease (based on an APRI score >1.5), and to have previous exposure to HCV treatment.

After adjustment, active PWID, low‐income, drinking alcohol and living in the province of Saskatchewan were associated with lower rates of DAA treatment initiation (Table 2). Indigenous peoples, women and non‐active PWID also tended to have lower treatment rates. Conversely, MSM were more likely to initiate DAAs as were people with significant liver fibrosis and controlled HIV viraemia. Stratified Cox models confirmed the results of the adjusted model summarized in Table 2 (results not shown).

**Table 2:** Predictors of second‐generation direct acting antiviral treatment initiation

	Unadjusted model HR (95% CI)	Adjusted model aHR (95% CI)
Age (per 10‐year)	1.60 (1.37, 1.87)	1.12 (0.93, 1.35)
Indigenous people	0.23 (0.14, 0.37)	0.70 (0.43, 1.15)
Sex (reference heterosexual men)		
Women	0.71 (0.48, 1.04)	0.85 (0.53, 1.36)
MSM	2.38 (1.74, 3.24)	1.95 (1.33, 2.86)
Injection Drug Use (reference non‐PWID)		
Active PWID^a^	0.26 (0.18, 0.40)	0.60 (0.38, 0.94)
Past PWID^b^	0.54 (0.39, 0.75)	0.88 (0.58, 1.33)
Income (<$18 000/year)	0.45 (0.34, 0.61)	0.50 (0.35, 0.71)
Alcohol use	0.96 (0.73, 1.27)	0.74 (0.58, 0.94)
Undetectable HIV viral load	2.55 (1.70, 3.83)	1.73 (1.20, 2.50)
Significant Liver Fibrosis (APRI > 1.5)	2.60 (1.94, 3.48)	2.28 (1.64, 3.16)
HCV genotype (reference genotype 1)		
2	1.21 (0.66, 2.24)	1.12 (0.57, 2.18)
3	0.59 (0.38, 0.92)	0.69 (0.42, 1.13)
4	2.48 (1.15, 5.22)	1.51 (0.66, 3.16)
Province of residence^c^ (reference British Columbia)		
Saskatchewan	0.02 (0.00, 0.17)	0.04 (0.01, 0.11)
Alberta/Ontario	1.00 (0.69, 1.44)	0.58 (0.24, 1.41)
Quebec	1.60 (1.15, 2.23)	1.52 (0.66, 3.51)

Adjusted model included all predictors listed in Table 2. Undetectable HIV RNA (RNA < 50 copies/mL).

HCV: hepatitis C virus; PWID: person who inject drugs; MSM: men who have sex with men; APRI: AST to platelet ratio index.

a Active PWID: Use of any injection drugs within 6 months of cohort visit (self reported).

b Past PWID: Not actively injecting drugs (as defined above) however exposure to injection drugs (self reported).

c Canadian province of residence (British Columbia, Saskatchewan, Alberta/Ontario and Quebec; based on DAA policy restrictions 27).

**Figure 2:**
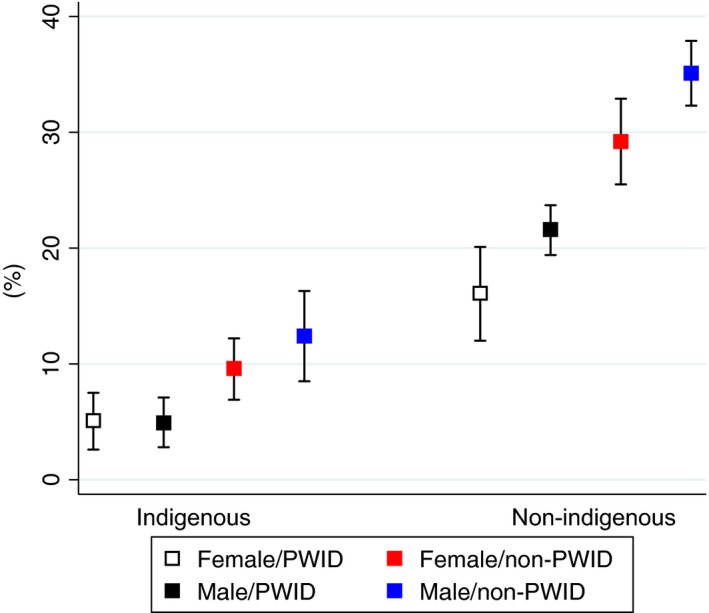
Two‐year probability of second‐generation DAA initiation by population profile. Probability (%), whiskers represent 95% confidence intervals. PWID: people who inject drug.

Figure 2 illustrates the 2‐year probability of initiating second‐generation DAA treatment by eight population profiles based on the multivariate Cox model. Across all three factors of interest (women, Indigenous peoples and active PWID) the clearest delineation in uptake exists between Indigenous peoples compared to other ethnicities. Among our cohort the profile with the lowest probability (5%, 95% CI 3–8%) of initiating second‐generation DAAs were female, Indigenous, PWID. Not having any of these risk factors resulted in a 35% (95% CI 32–38%) probability of initiating DAA treatment. Table S2 summarizes the unadjusted initiation rates per 100 person‐years by key population groups.

As these risk factors may occur together, we attempted to isolate which was most responsible for lower rates of DAA initiation by creating a hypothetical cohort with fixed characteristics of those more likely to initiate treatment. As illustrated in Figure S2, it appears that IDU drove most of the effect although being Indigenous and a woman additionally contributed to the lower probability of initiating DAAs.

### 3.3 Second‐generation DAA treatment response

**Figure 3:**
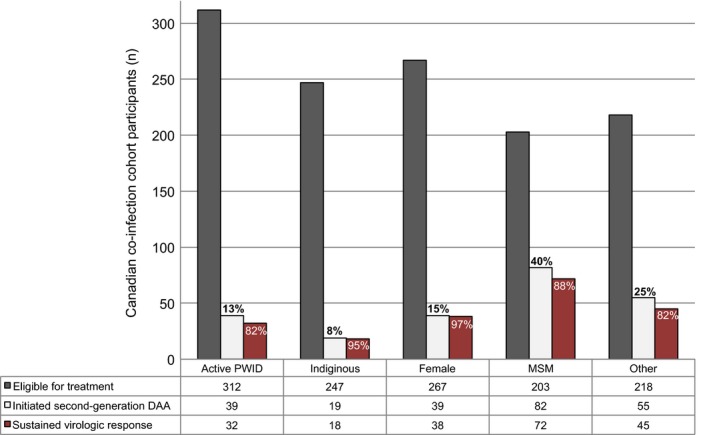
Second‐generation DAA treatment cascade. Bar graph represents overall numbers of patients eligible for treatment, initiate second‐generation DAAs and achieved SVR by key populations. CCC: Canadian Co‐Infection Cohort; PWID: people who inject drugs; MSM: men who have sex with men; SVR: sustained virologic response.

Figure 3 illustrates the cascade of HCV treatment among CCC participants who were eligible, initiated and achieved SVR. Overall, SVR rates were 87% (176/202). By definition, 26 people were classified as non‐responders (null (n = 8), breakthrough (n = 6), partial response (n = 4), deaths (n = 3), relapse (n = 1) and missing post treatment HCV RNA (n = 4)). Despite low treatment uptake among the key populations of interest, SVR rates were high: 82% in active PWID (32/39, 1 missing), 90% among Indigenous peoples (18/19*),* 97% among women (38/39) and 88% (72/82) in MSM. For comparative purposes, a category defined as “*other*” was created to include populations who were not Indigenous, women, active PWID or MSM. Again, although this group had a higher rate of initiation, SVR rates (82%; 45/55, 3 missing) were similar to the overall cohort. No clear associations were observed between non‐response and clinical characteristics or specific treatment regimens.

## 4 Discussion

The development of DAAs has generated enthusiasm that HCV can be eliminated. However, the gap between near 100% curative treatments and viral elimination is immense if only a small segment of the population initiates treatment 41. HCV treatment cascades among HCV mono‐infected individuals highlight the need for better screening, diagnosis and linkage to care to ultimately cure HCV 23, 42, 43. HIV‐HCV coinfected populations are generally well identified and already engaged in HIV care therefore easier to reach compared to HCV mono‐infected populations. In a publicly funded healthcare setting with no overt restrictions limiting DAA uptake by socio‐demographic or behavioural factors, we found significant disparities existed among key HIV‐HCV co‐infected populations engaged in care. Although HCV treatment uptake was rapid after second‐generation DAA were approved, the MSM population largely drove this trend. In contrast PWID, and more generally, people of lower SES were far less likely to initiate treatment. Despite low treatment uptake in some groups, SVR rates were high in all key populations. Results from this study suggest that despite the advent of highly efficacious and well‐tolerated second‐generation DAA therapies, patient‐, system‐ and provider‐ barriers may still remain for many HIV‐HCV co‐infected populations.

### 4.1 Patient‐level barriers

In high‐income countries, HIV‐HCV co‐infection affects marginalized populations who are often socially disenfranchised with many competing priorities. Lower SES, substance abuse and mental illness have previously been associated with barriers to accessing healthcare 17, 20, 44. Results from our study provide evidence that patient‐related factors (IDU, low income and alcohol use) remain barriers to HCV treatment initiation in the DAA era. Disparities are also evident in Indigenous compared to non‐Indigenous individuals. High rates of IDU, predominantly among young Indigenous people, have recently increased rates of co‐infection significantly in Canada, specifically in the province of Saskatchewan 33. Similarly in Australia newly diagnosed HCV among Aboriginal people has increased by 38% from 2010 to 2014; in contrast during the same time period, notification rates among non‐Aboriginal people has decreased by 15% 45. While in Canada there are no system‐level barriers that limit treatment of PWID, our results suggest active PWID, and to a certain extent, past PWID are not accessing DAAs at the same rate as non‐PWID. Modelling studies have shown treating PWID to be cost‐effective, because treatment may also acts as prevention 14, 22. Furthermore, women face unique barriers to accessing treatment and care. Among women who inject drugs, reasons for not accessing healthcare and treatment may include child‐bearing, child care responsibilities, ongoing sex work, higher rates of mental health issues and lower access to harm‐reduction programs 46.

HIV+ MSM form an emerging risk group for HCV acquisition 47. We found MSM were far more likely to initiate treatment suggesting that broad treatment in this group is possible and could result in reduced HCV transmission. MSM in our cohort were more likely to have higher income and be more educated, and were less likely to inject drugs—all factors associated with initiating DAAs.

### 4.2 System‐level barriers

The extraordinary cost of DAAs has led many countries to restrict access to DAAs based on a variety of factors. Compared to the multi‐payer system in the United States where considerable variation in DAA coverage exists, specifically in regards to active substance use, coverage policies across Canada are more homogeneous. Although all Canadian citizens and permanent residents have insurance coverage for in‐hospital and physician services, medication coverage varies across the 10 provinces and three territories, with a mix of both public and private sources of insurance depending on individual characteristics. For example, people on social assistance receive public coverage for medications with no or minimal co‐payments and Indigenous people receive medication coverage from the First Nations and Inuit Health Branch (FNIHB). During the period of study, all provinces and territories in Canada, with the exception of Quebec, restricted the reimbursement of DAAs to those with advanced liver fibrosis (F2 or greater) 27. Consistent with this, the strongest predictor of treatment initiation in our study was having advanced liver fibrosis. In addition, DAA initiation varied by province; for example, a larger proportion of co‐infected individuals were treated in Quebec compared to other provinces. Quebec was the first province to reimburse simeprevir and sofosbuvir with no liver fibrosis restrictions (in 2014) and later introduced a tiered reimbursement strategy that allowed all co‐infected individuals access to ledipasvir/sofosbuvir and paritaprevir/ombitasvir/dasabuvir regardless of liver fibrosis stage. Even with Quebec's more inclusive insurance coverage of DAAs, PWID compared to people not reporting IDU were still less likely to initiate treatment, whereas MSM and people with advanced liver fibrosis were more likely to initiate treatment (data not shown). In contrast, people residing in Saskatchewan initiated DAAs at a significantly lower rate than in other provinces. In Saskatchewan patients tended to be younger PWID with less advanced liver disease illustrating how even though significant liver fibrosis requirements may seem like, a non‐discriminatory policy restriction; it may still lead to social and health inequities.

### 4.3 Provider‐level barriers

Providers are faced with the challenge of managing clinically and socially complex co‐infected patients and navigating administrative hurdles to access treatments. We found HCV genotypes were missing for 10% of our cohort, indicating that even though engaged in care, such people were not being considered for treatment. Those with unknown genotypes were more likely to be PWID and Indigenous. Even though IDU has been characterized as a chronic relapsing brain disease, PWID may continue to face stigma and discrimination from health professionals 48. It is also possible providers may have concerns about poor adherence and reinfections among PWID 17, 32, 49. Based on successful HCV treatment trials and economic analyses, international guidelines now recommend that treating PWID should be made a priority 50, 51. We found similar SVR rates in active PWID compared to non‐PWID in a real‐world setting, further supporting international guidelines to treat PWID.

Previous published reports exist on DAA treatment disparities using data from the Veterans Affairs (VA) and TRIO Network cohorts 52, 53. In the VA cohort, black patients and younger women were less likely to initiate DAA treatment 52. However it is difficult to generalize results from the VA cohort to other healthcare systems since this cohort is primarily male and has broader access to healthcare and HCV treatment compared to other American cohorts 54, 55. The TRIO network compared receipt of DAAs according to type of insurance providers (Medicaid or commercial) and, as in other studies, found that Medicaid prescribers faced more barriers to treatment due to processes related to insurance coverage and financial reasons 29, 53, 56. Our study focuses specifically on HIV‐HCV co‐infected individuals, a unique population that arguably stands to benefit the most from HCV viral clearance 35, 57, 58. We used data from a representative, prospective cohort of co‐infected individuals already engaged in care that included active and past PWID, women and Indigenous peoples. Furthermore, patient characteristics and treatment information were based on prospective data collection and not secondary data extraction from billing codes. Most recently Janjua et al. 59 described shifts in the characteristics of people who received interferon‐based HCV regimens compared to DAAs, using a population‐based cohort in British Columbia, Canada and found HIV‐HCV co‐infected individuals were more likely to initiate DAA treatment compared to the interferon era. Results from our study highlight the heterogeneity of the HIV‐HCV co‐infected population and the importance of evaluating uptake among specific key populations.

Our study has limitations. Overlapping patient‐level barriers make it difficult to identify independent reasons for treatment disparities and due to our sample size it was not possible for us to explore formal statistical tests to identify synergistic relationships between Indigenous ethnicity, IDU and/or sex. Having supplemental healthcare insurance coverage (third party private insurance) maybe another important predictor of treatment initiation, however not routinely collected. Although the vast majority of this cohort was making less than $18 000/year therefore qualified for provincial drug assistance. Furthermore, four people (2%) who initiated treatment had a missing treatment response. This could mean the overall SVR rates may be underestimated, if in fact the missing responses were undetectable. Finally, we focused on a population already in care ‐ that is, at the end of the cascade of care. To evaluate the population level impact of DAAs, it will be important to evaluate each step of the care continuum, including ongoing surveillance of reinfections. Close follow‐up to document treatment response and reinfections will be important as treatments are rolled out more broadly.

## 5 Conclusions

In this study, we found important disparities in DAA uptake existed among key HIV‐HCV co‐infected populations already engaged in care in a publicly funded healthcare system, in particular PWID and more generally people of low SES. Low rates of treatment cannot be justified based on SVR rates, which were relatively high in all sub‐groups. Availability of generics in developing countries and recent pricing agreements in developed countries should mean wider access to these curative therapies in the near future. However, if patient‐level barriers are not addressed, even in high‐income countries, we will fail to make headway in reaching HCV elimination targets set out by the WHO by 2030. The next steps will be to develop targeted interventions that can be ultimately scaled‐up to address unique patient‐level barriers and to educate providers and policy makers to reduce stigma against treating key coinfected populations worldwide.

## Competing interests

None of the authors feel in conflict of interest with regards to this study and there was no pharmaceutical industry support to conduct this study. Sahar Saeed, Erin C Strumpf, Erica Moodie, Jim Young and Roy Nitulescu have no conflicts of interest to declare. Marina Klein has received research grants for investigator‐initiated trials from Merck and ViiV Healthcare; consulting fees from ViiV Healthcare, Bristol‐Meyers Squibb, Merck, Gilead and AbbVie. Joseph Cox received consulting fees from Bristol‐Meyers Squibb; grants from ViiV Healthcare and Gilead; and payment for lectures from Merck. Alex Wong received consulting and honoraria from Merck, Gilead Sciences, Bristol Myers Squibb, Pfizer, Janssen, Boehringer‐Ingelheim and AbbVie also funding for regional and provincial programming was received from Merck, Gilead Sciences, Bristol Myers Squibb, ViiV, Janssen and AbbVie. Sharon Walmsley received grants, consulting fees, lecture fees, nonfinancial support and fees for the development of educational presentations from Merck, ViiV Healthcare, GlaxoSmithKline, Pfizer, Gilead, Abbvie, Bristol‐Myers Squibb and Janssen. Curtis Cooper reports consulting fees from AbbVie, Gilead and Merck; and grants from AbbVie and Gilead. Marie‐Louise Vachon has received consulting fees from Boehringer Ingelheim and Merck; consulting fees and lecture honoraria from Janssen Pharmaceuticals, Gilead, Hoffman–La Roche and Vertex Pharmaceuticals; and speaker fees from Gilead. Valérie Martel‐ Laferrière reports consulting fees from Merck and Gilead; grant from Gilead; and lecture fees from AbbVie, Merck and Gilead. Mark Hull, received grant support from National Institute on Drug Abuse (NIDA R01DA031043‐01). Honoraria were received from (speaking engagements and/or consultancy) AbbVie, Bristol Myers Squibb, Gilead, Merck, Ortho‐Janssen and ViiV. Brian Conway reports grants, travel support, personal fees for speakers bureau and advisory board participation from AbbVie, Gilead and Merck.

## Funding

This work was supported by the Fonds de recherche du Québec –Santé (FRQ‐S); Réseau SIDA/maladies infectieuses, the Canadian Institutes of Health Research (CIHR FDN 143270) and the CIHR Canadian HIV Trials Network (CTN222). Sahar Saeed is supported by doctoral awards from Canadian Institutes of Health Research and the Canadian Hepatitis C Network. Erin C Strumpf and Erica Moodie are supported by a Chercheur boursier Junior 2 from the FRQ‐S. Marina B Klein is supported by a “Chercheurs Nationaux” career award from the FRQ‐S.

## Supporting information


**Figure S1.** Eligible Cohort Flow Diagram.
**Figure S2.** Two‐Year Probability of DAA Second Generation DAA Initiation (Fixed Covariates).
**Table S1.** Demographics of Participants excluded from study population (as illustrated by Flow Diagram).
**Table S2.** DAA second generation DAA initiations by population profile (raw data).
