# Supplementary material for: Disparities in direct acting antivirals uptake in HIV‐hepatitis C co‐infected populations in Canada
Source: J Int AIDS Soc. 2017 Nov 8;20(3):e25013. doi: 10.1002/jia2.25013 (PMC5810331; doi:10.1002/jia2.25013)
Supplement: Supplementary file 1 — Figure S1. Eligible Cohort Flow Diagram. Figure S2. Two‐Year Probability of DAA Second Generation DAA Initiation (Fixed Covariates). Table S1. Demographics of Participants excluded from study population (as illustrated by Flow Diagram). Table S2. DAA second generation DAA initiations by population profile (raw data). [file JIA2-20-e25013-s001.docx]

**Supplemental Figure1. Participant Flow Diagram**

**Canadian HIV-HCV**

**Co-Infection Cohort**

2002-2016

**(N=1699)**

**Excluded (N=766)**

Lost to follow (N=160)

(*Last Study Visit <Nov 20 2012)*

Died (N=149)

Withdrew (N=75)

HCV RNA Negative (N=382)

**Active Participants**

(Nov 21, 2013-Dec 31, 2015**)**

**(N=933)**

Eligible Cohort

**(N=911)**

SVR: 176/202=87%

Non-Response**: 26/202= 13%

2^nd^ Generation DAAs

**(N=199)***

**202 initiations**

Did Not Initiate

**(N=712)**

**Excluded (N=22)**

Initiated DAAs through a

Clinical Trial

**Supplemental Table 1. Participants excluded from study population (as illustrated by Flow Diagram)**

|  | **Left Censored**  **(Lost to follow up/ Withdrew)**  No. (%) **N=235** | **Clinical Trial**  **Participant**  No. (%) **N=22** |
| --- | --- | --- |
| Age (years, IQR) | 45 (39, 51) | 51 (47, 55) |
| Indigenous | 37 (16%) | 3 (14%) |
| Women | 56 (24%) | 8 (36%) |
| MSM | 49 (21%) | 5 (23%) |
| Active PWID | 68 (29%) | 3 (14%) |
| Past PWID | 119 (51%) | 15 (68%) |
| Income (<$18,000/year) | 176 (75%) | 15 (68%) |
| Alcohol Use | 122 (52%) | 11 (50%) |
| Undetectable HIV Viral Load (<50 copies/mL) | 150 (64%) | 19 (86%) |
| Significant Liver Fibrosis (APRI>1.5) | 39 (17%) | 22 (100%) |
| HCV Genotype  1  2  3  4  Missing | 131 (56%)  9 (4%)  30 (13%)  7 (3%)  58 (25%) | 18 (82%)  1 (5%)  2 (9%)  1 (5%)  0 |
| Province of Residence  British Columbia  Alberta  Ontario  Quebec | 72 (31%)  7 (3%)  56 (24%)  99 (42%) | 9 (41%)  0  8 (36%)  5 (23%) |

Undetectable HIV RNA (RNA<50 copies/mL) HCV- Hepatitis C Virus; PWID-Person who Inject Drugs; MSM-Men who have Sex with Men; APRI- AST to **P**latelet **R**atio Index

**Supplemental Table 2. DAA Second Generation DAA Initiations by Population Profile (Raw data)**

|  |  | **Number of Initiations (numerator)** | **Total number of people** | **Total follow-up time (years)** | **Rates per 100 person-years** |
| --- | --- | --- | --- | --- | --- |
| Indigenous | Female/PWID | 3 | 49 | 98 | 3 |
|  | Male/PWID | 1 | 56 | 114 | 1 |
|  | Female/non-PWID | 5 | 64 | 131 | 3 |
|  | Male/non-PWID | 5 | 51 | 96 | 5 |
| Non-Indigenous | Female/PWID | 4 | 45 | 87 | 5 |
|  | Male/PWID | 18 | 113 | 206 | 9 |
|  | Female/non-PWID | 27 | 101 | 87 | 31 |
|  | Male/non-PWID | 54 | 204 | 180 | 30 |

**Supplemental Figure 2. Two-Year Probability of DAA Second Generation DAA Initiation (Fixed Covariates)**
